# Dhat syndrome: A case report on a culture-bound challenge

**DOI:** 10.1192/j.eurpsy.2021.1941

**Published:** 2021-08-13

**Authors:** L. Ilzarbe, D. Ilzarbe, N. Arbelo, C. Llach, G. Anmella, E. Vieta, A. Murru

**Affiliations:** 1 Department Of Psychiatry And Psychology, Institute Of Neuroscience, Hospital Clinic de Barcelona, Barcelona, Spain; 2 Department Of Child And Adolescent Psychiatry And Psychology, Idibaps, University Of Barcelona, Hospital Clínic de Barcelona, Barcelona, Spain; 3 Department Of Child And Adolescent Psychiatry, King’s College London, London, United Kingdom; 4 Bipolar And Depressive Disorders Unit, Idibaps Cibersam, Hospital Clinic, University Of Barcelona, Hospital Clínic de Barcelona, Barcelona, Spain

**Keywords:** Culture, dhat syndrome, transcultural psychiatry, Migration

## Abstract

**Introduction:**

Dhat Syndrome is a culture-bound entity frequent in the Indian subcontinent. It is characterized by somatic symptoms, together with depressive and anxiety features, specifically focused on the belief of losing semen through urine^1^.

**Objectives:**

To describe an atypical Dhat Syndrome case in European cultural context,and to determine the appropriate diagnostic frame and subsequent therapeutic approach.

**Methods:**

We present the case of a 37-year-old Indian man attended in our psychiatric outpatient unit mainly due to somatic complaints (gastrointestinal, sexual dysfunction, weakness, and dizziness). He interpreted his problem as possibly due to diabetes and hypothyroidism, and specifically from sugar loss in urine. Organic diseases were excluded.

**Results:**

Although considered as culture-bound, Dhat syndrome has been classified as a subtype of depression, anxiety disorder, somatoform disorder^2,3,4^, and even a prodromal phase of schizophrenia^5^. Antidepressants and benzodiazepines are the most recommended pharmacological treatments^1^. Antipsychotic agents have been used when clear psychotic symptoms were present (auditory hallucinations,delusions)^5^. Nonetheless, paliperidone 6mg/d was initiated at baseline, with good response and partial remission of the symptoms at two weeks, despite the absence of clear psychotic features. Culturally-informed cognitive-behavioural therapy, as well as sexual education could be beneficial were planned and initiated^1^.

**Conclusions:**

Data on Dhat Syndrome is scarce, yet agreement exist in considering the cultural context of the patient to avoid diagnostic delays. The adequate treatment remains uncertain. Antipsychotics may be a potential treatment. Further research is necessary to clarify the nature of this syndrome but European clinicians must be aware of culturally-mediated psychiatric manifestations which are increasingly prevalent due to globalization.

**Disclosure:**

No significant relationships.

